# Evaluation of commercial RNA extraction kits for long-read metatranscriptomics in soil

**DOI:** 10.1099/mgen.0.001298

**Published:** 2024-09-19

**Authors:** Daniel G. Barber, Christian A. Davies, Iain P. Hartley, Richard K. Tennant

**Affiliations:** 1 Geography, Faculty of Environment, Science and Economy, Amory Building, Rennes Drive, Exeter, Devon, EX4 4RJ, UK; 2 Shell International Exploration and Production Inc., Shell Technology Centre Houston, Houston, TX, 77082, USA

**Keywords:** kit comparison, metatranscriptomics, microbiome, Oxford Nanopore, RNA, soil

## Abstract

Metatranscriptomic analysis of the soil microbiome has the potential to reveal molecular mechanisms that drive soil processes regulated by the microbial community. Therefore, RNA samples must be of sufficient yield and quality to robustly quantify differential gene expression. While short-read sequencing technology is often favoured for metatranscriptomics, long-read sequencing has the potential to provide several benefits over short-read technologies. The ability to resolve complete transcripts on a portable sequencing platform for a relatively low capital expenditure makes Oxford Nanopore Technology an attractive prospect for addressing many of the challenges of soil metatranscriptomics. To fully enable long-read metatranscriptomic analysis of the functional molecular pathways expressed in these diverse habitats, RNA purification methods from soil must be optimised for long-read sequencing. Here we compare RNA samples purified using five commercially available extraction kits designed for use with soil. We found that the Qiagen RNeasy PowerSoil Total RNA Kit performed the best across RNA yield, quality and purity and was robust across different soil types. We found that sufficient sequencing depth can be achieved to characterise the active community for total RNA samples using Oxford Nanopore Technology, and discuss its current limitations for differential gene expression analysis in soil studies.

**Impact Statement**

Global agricultural soils have lost more than 100 billion tonnes of carbon. Our understanding of how soil carbon storage is controlled has advanced substantially in recent years, with the physical and chemical processes that promote long-term persistence of soil organic matter starting to be identified. While soil metagenomics has become more commonplace, soil metatranscriptomics, the analysis of RNA, and therefore the molecular functionality being expressed within a microbiome, is still in its infancy due to a range of factors including, the difficulty in purifying sufficient RNA from soil with a concentration and quality suitable for RNA sequencing. This research solves the methodological challenges associated with purifying sufficient RNA for soil metatranscriptomics and demonstrates the potential of long-read sequencing for elucidating soil microbial processes. Furthermore, this study provides a foundation in the pursuit of improving our understanding of the soil molecular mechanisms responsible for carbon sequestration.

## Data Summary

The datasets generated during the current study are available in the NCBI Sequence Read Archive repository, PRJNA1079547.

## Introduction

Next generation sequencing (NGS) has become a foundational technology for investigating functional microbiology [1] and characterising microbial communities found in a range of environments [2, 3, 4]. Application of NGS to soil samples has become commonplace, with an increase in the number of articles that employ amplicon sequencing for characterisation of the microbiome [5]. Metabarcoding is a cost effective and well-established tool for microbial community analysis and furthermore, bioinformatic tools have been developed to predict metabolic functionality based on amplicon sequence data from metagenomic samples [6]. These *in silico* methods offer the ability to predict how a particular microbiome is functioning. However, employing these bioinformatic tools on complex environmental samples is limited by the inclusion of incorrectly predicted genes and the exclusion of many genes found in whole genome sequence data [7]. Similarly, while function can be derived from whole genome sequence data by analysing the presence of gene pathways [8], active expression cannot be directly quantified.

Metatranscriptomics is the study of gene expression within a biological community and has the potential to analyse the functionality of the soil microbiome directly by sequencing gene transcripts [9]. In the soil environment, metatranscriptomics has been applied to the study of microbial responses to climate change [10], the application of an agricultural amendment [11] and has been used to elucidate the fundamentals of plant-microbe interactions [12]. Compared to metagenomic studies, there are comparatively few metatranscriptomic studies conducted on soil environments [13], with low-yield, low-quality samples often cited as the rationale for unsuccessful sequencing [11]. These challenges may be soil-type dependent, a function of microbial load, or change with the relative activity of different microbial communities [14]. Long-read sequencing offered by Oxford Nanopore Technology (ONT) is not often adopted as a tool for metatranscriptomics with the highly accurate short read Illumina technology usually favoured [10, 15, 16]. However, the long-read sequencing platform of ONT may provide multiple benefits if it can be optimised for soil samples. The ability to sequence full-length transcripts could remove, or at least improve, the assembly step required with short-read transcriptomics [17]. Longer reads are also more accurately classified prior to assembly as there are more contiguous base pairs available for alignment [18]. In addition ONT platforms are highly portable facilitating in-field sequencing, a potentially crucial factor for reducing the impact of sample storage on the dynamic metatranscriptome [19]. The ONT platform has a much lower capital cost than its short-read alternatives, with a MinION starter pack costing less than £2000 [20]. This low CAPEX makes ONT sequencing accessible to more research laboratories and enables soil community analysis on a wider variety of global soils.

Efforts have been made to provide efficient ‘in-house’ RNA extraction methods for soil samples optimised for ONT sequencing, with the cost of commercial RNA extraction kits and the ability to optimise extraction buffers for different soil types cited as the advantages for customised methods [21]. These tailored methods yield good quality RNA, suitable for sequencing using the ONT direct RNA kits at adequate sequencing depth for metatranscriptomic analysis with an average read length of 702 base pairs. However, currently the direct RNA sequencing technology provided by ONT does not support multiplexing meaning that only single samples can be sequenced per sequencing flow cell, dramatically increasing the cost and time incurred to generate sufficient data. In addition, the newest direct RNA sequencing kit, SQK-RNA004, and RNA flow cells, FLO-PRO004RA, require significantly greater RNA concentrations for optimum sequencing than previous iterations making this method prohibitive for many RNA samples extracted from soil. Furthermore, there is currently a lack of standardisation in methods of sampling and sequencing for soil metatranscriptomic studies and as such, commercially available RNA extraction kits designed for soil may provide greater replicability between laboratories [22].

Here we investigate the effectiveness and impact of five commercial soil RNA extraction kits on the RNA yield, integrity and purity from soil. We assess how these metrics affect the generation of classifiable sequence data that are suitable for downstream bioinformatic analyses. We then evaluate the consistency across different soil types to determine the most efficient commercially available soil RNA extraction kit for metatranscriptomic studies.

## Methods

### Soil collection and incubation

Approximately 2 kg of soil was sampled from a depth of 0–20 cm at a pasture site used for the grazing of a small flock of approximately 20 sheep in Nadderwater, Devon, England (50.731023, –3.582804) during February 2023. This soil was used for the purposes of comparing RNA extractions conducted using five different RNA extraction kits on replicate soil samples. Soil was sealed in two polyethylene bags, protected from light, and incubated at room temperature for 1 week before subsampling. Soil was incubated in this way to encourage higher microbial activity in the samples due to soil being collected during the winter months in South West England. After incubation, soil was shaken in the double sealed bags to homogenise the sample as much as possible before approximately 50 g of soil was removed. The 50 g of soil was thoroughly mixed again before subsampling into 2 ml microcentrifuge tubes for input weights of up 0.5 g or 15 ml centrifuge tubes for up to 2 g of input. Subsample weights were set at the maximum input level recommended by the manufacturer for each extraction kit. Then 2 ml g^−1^ of Lifeguard soil perseveration solution (Qiagen, Germany) was then added to each tube and samples resuspended by vortexing. Samples were stored at −80 °C until extractions were conducted. To account for differences in incubation and storage time, subsampling was conducted in batches. Two batches of six samples were prepared after an incubation time of 7 and 28 days respectively, storage time at −80 °C varied between kits for each batch. To test the robustness of the best performing extraction kit on different soil types, soil samples were collected from an arable field (50.68772, –3.31217), a heathland (50.68523, –3.326668), a permanent pasture (50.68029, –3.29261) and a deciduous woodland (50.65239, –3.34743) within the Clinton Estate in Devon, England, and stored after collection at 4 °C for up to 30 days. Samples were sieved at 2 mm and roots were removed. Then 30 g was subsampled into pots and incubated at room temperature for 5 days before RNA extraction.

### RNA extraction

RNA extractions were performed following the manufacturers guidelines, unless described below, using the FastRNA Pro Soil-Direct Kit (MP Biomedicals, California, USA), *Quick*-RNA Faecal/Soil Microbe Microprep kit (Zymogen Research, California, USA), NucleoBond RNA Soil Mini kit for RNA from soil (Machery-Nagel, Germany), NucleoBond RNA Soil Midi kit for RNA from soil (Machery-Nagel, Germany) and RNeasy PowerSoil Total RNA kit (Qiagen, Germany). Lysis steps for each extraction were conducted using the FastPrep-24 5G homogeniser (MP Biomedicals, California, USA) according to the manufacturer’s instructions for the respective extraction kit. A 100 µl elution volume was used for each kit as specified by the manufacturer except the *Quick*-RNA Faecal/Soil Microbe Microprep kit for which a 10 µl elution volume was used. Samples were thawed on ice and centrifuged at 4 000 rcf for 10 mins before removing all preservation solution. The samples were then resuspended in the appropriate lysis buffer and transferred into lysis tubes to begin the extraction. RNase Zap (Thermo Scientific, Massachusetts, USA) was used throughout each extraction to clean working surfaces and gloves. The order in which each kit was used for extraction over the experimental period was randomised between batches. RNA was extracted from Clinton Estate soils using the RNeasy PowerSoil Total RNA kit from 2 g of fresh soil immediately after sampling. Acronyms used in this study for each kit are detailed in Table 1.

**Table 1.** RNA extraction kits used in this study

Extraction kit	Product code	Acronym used in this study	Input wt (g)	Homogenisation speed (m s^−1^)	Homogenisation time (seconds)	Elution vol.(µl)
FastRNA Pro Soil-Direct Kit (MP Biomedicals)	6 070 050	MP	~0.5	6	40	100
*Quick*-RNA Faecal/Soil Microbe Microprep kit (Zymogen Research)	R2040	Z	~0.25	6	40	10–15
NucleoBond RNA Soil Mini kit for RNA from soil (Machery-Nagel)	740 142.50	Nm	~0.5	6	40	100
NucleoBond RNA Soil Midi kit for RNA from soil (Machery-Nagel	740 140.20	Nd	~2	6	40	100
RNeasy PowerSoil Total RNA kit (Qiagen)	12866–25	Q	~2	6	40	100

### Quantification of RNA yield and quality

RNA concentrations were quantified using the Qubit high sensitivity (HS) and broad range (BR) RNA assay (Thermo Scientific, Massachusetts, USA) in conjunction with the Qubit Flex fluorometer (Thermo Scientific, Massachusetts, USA). Total RNA was calculated by multiplying RNA concentration by the corresponding elution volume. Extraction efficiency was calculated by dividing total RNA by the input weight of soil in the extraction. Fragment length distribution and RIN^e^ were calculated using the Agilent Tapestation 4200 (Agilent Technologies, California, USA) using RNA or high sensitivity RNA tapes, depending on the concentration of the RNA sample. Sample purity was determined by the 260 : 280 nm absorbance ratio using the BMG LABTECH LVis plate and Fluorostar spectrophotometer (BMG LABTECH, Germany). MARS software (BMG LABTECH, Germany) was used to calculate 260 : 280 nm absorbance ratio where concentrations were in the range for the sensitivity of the instrument. A NanoPhotometer NP80 (IMPLEN, Germany) was used for determining purity for RNA samples which had concentrations below 20 ng µl^−1^.

### DNase treatment

DNA contamination was quantified by Qubit BR dsDNA assay in conjunction with the Qubit Flex fluorometer. The turbo DNA-*free* kit (Invitrogen, California, USA) was used for the removal of DNA contamination from RNA samples. The Routine DNase treatment protocol was followed according to the manufacturer’s instructions with an enzyme incubation time of 30 min.

### Library preparation and sequencing

Reverse transcription was performed using Maxima H Minus Reverse transcriptase (Thermo Scientific, Massachusetts, USA) according to the manufacturer’s instructions. RNA sequencing libraries were prepared using SQK-PCB111 (Oxford Nanopore Technologies, UK). Prepared libraries were sequenced using a R9.4.1 MinION or PromethION flow cells coupled to either a GridION or the PromethION P2 solo sequencing device. Basecalling was performed using Guppy v6.5.7 using the High accuracy basecalling model.

### Data analysis

Adapter trimming and demultiplexing was performed using porechop v.0.2.4 [23]. Taxonomic classification of adapter trimmed reads was conducted using centrifuge v.1.0.4 [24] against a custom database containing RefSeq complete genomes (November 2023) for all available bacteria, archaea, viruses, fungi, protozoa, plants, and vertebrate mammals. All adapter trimmed reads were also filtered using sortmerna [25] v4.3.6 using the smr_v4.3_default_db.fasta provided with software as the database for rRNA alignment. Reads sorted as ‘other’ by sortmerna were classified using DIAMOND [26] v2.1.7 against the NCBI nr database (November 2023). Count tables and KEGG [27] mappings were produced using MEGAN6 Ultimate edition [28]. Species richness, Bray-Curtis dissimilarity and ADONIS2 results were produced with the R package vegan [29] v2.6–4. ANOVA and Tukey’s multiple comparisons tests were performed in graphpad prism v10.1.1.

## Results

### Assessment of commercial RNA extraction kits

**Fig. 1.**
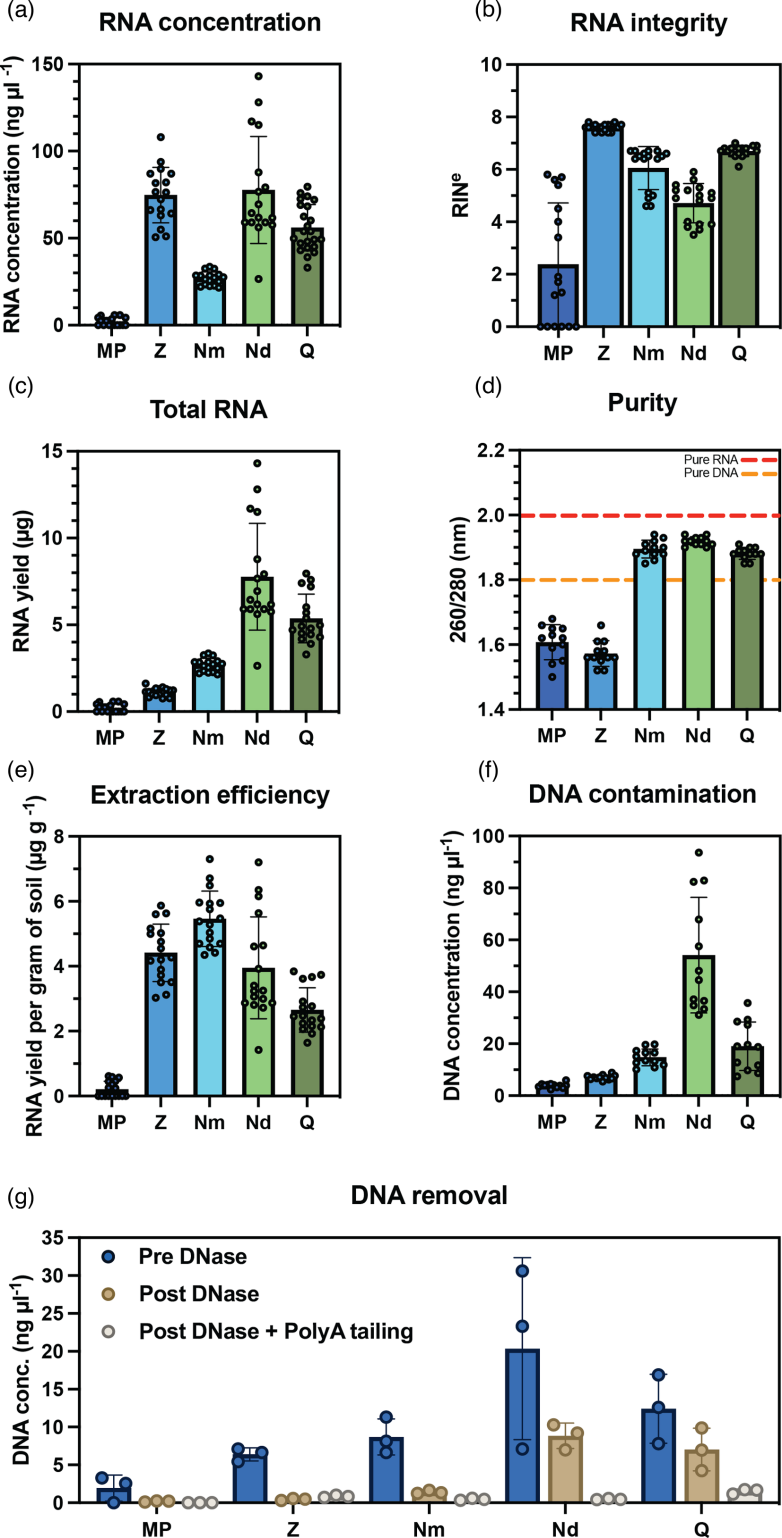
Comparison of RNA extracted from soil using five commercially available kits. RNA concentration (**a**) and RNA integrity (**b**) was quantified for each kit, MPBio (MP), Zymo (Z), Nucleobond Mini (Nm), Nucleobond Midi (Nd), and Qiagen (Q). Total RNA (**c**) was calculated as concentration multiplied by elution volume. Each kit was assessed the purity of extracted RNA (**d**), extraction efficiency (**e**) and DNA contamination (**f**). The effectiveness of DNase treatment was assessed for a subset of all samples (**g**). Error bars represent SD. *n*≥12 (**a-f**), *n*=3 (**g**).

RNA concentration, total RNA yield, and RNA yield per gram of soil was calculated for each extraction kit (Fig. 1a, c and e). The NucleoBond Midi (Nd) kit yielded an average of 78 ng µl^−1^, Zymo (Z) 75 ng µl^−1^, Qiagen (Q) 56 ng µl^−1^, Nucleobond Mini (Nm) 27 ng µl^−1^ and 2 ng µl^−1^ of RNA was purified using the MPBio (MP) kit. A one-way ANOVA and subsequent Tukey multiple comparison test showed there was a significant difference in RNA concentrations extracted from each kit (*P*<0.01) except between the Z and Nd kits. The highest total RNA yield was achieved from the Nd and Q kits which had an input weight of 2 g. RNA yield per gram of soil gives an indication of the relative efficiency with which each kit can extract RNA from a soil sample (Fig. 1e). The Nm kit yielded 5.5 µg g^−1^, the highest concentration of the kits tested, in contrast the MP kit was only able to extract 0.21 µg g^−1^.

RNA integrity was quantified using RIN^e^ (Fig. 1b). The MP kit had the lowest average RIN^e^ score of 3.3 while the Z kit produced the highest average RIN^e^ of 7.7. The Nd kit had a comparatively low average RIN^e^ score of 4.4. No significant differences were found between the RIN^e^ scores of the Q and Z kits, both performing well with an average RIN^e^ of greater than 7. RNA purity score was calculated by measuring the 260 : 280 nm absorbance ratio (Fig. 1d), with pure RNA having a 260 : 280 nm ratio of 2.0. Purity scores of greater that 1.85 were observed for the Nm, Nd and Q kits while purity values of below 1.6 were measured for the MP and Z kits. DNA contamination was quantified for each kit (Fig. 1f) and shows the Qiagen kit produces comparatively low DNA contamination, 20 ng µl^−1^, when compared to the Nd kit which has the same 2 g sample input, indicating the Qiagen kit is more effective of purifying RNA during the extraction process. Removal of DNA from the sample prior to PolyA tailing of transcripts is necessary for RNA sequencing library preparation. The effectiveness of DNase treatment for the removal of DNA contamination was tested on the purified RNA samples (Fig. 1g). DNA concentrations were measured before and after DNase treatment, and after PolyA tailing and subsequent bead purification. Using the least rigorous protocol detailed by the manufacturer, effective removal of DNA contamination was observed for all samples after PolyA tailing with <1.5 ng µl^−1^ across the kits. Table 2 provides a summary of each of the kits effectiveness on purifying RNA that was suitable for long-read RNA sequencing library preparation and their handling and consistency during extraction.

**Table 2.** Scoring of commercial RNA extraction kits used in this study. Sufficient yield refers to the ability of each kit to purify RNA at adequate concentrations for ONT library preparation without the need for further processing, - indicates insufficient yield, + indicates sufficient yield for library preparation, ++ indicates yield sufficient for multiple library preparations. RNA Integrity, the ability of each kit to purify intact full length transcripts, score based on RIN^e^ score, - indicates a RIN^e^ of <5, + indicates RIN^e^ 6–7, ++ indicates RIN^e^ > 7. Purity, - indicates 260:280 <1.8, + indicates 260:280 between 1.8–2.0. Extraction handling and process refers to the ease, and repeatability of results from each kit, qualitatively, -- indicates a protocol process that adversely effects the quality of purified RNA, - indicates a difficult protocol to achieve consistent results, + indicates a user-friendly protocol.

Extraction kit	Acronym used in this study	Sufficient yield	RNA integrity	Purity	Extraction handling & process
FastRNA Pro Soil-Direct Kit(MP Biomedicals)	MP	−	−	−	−
*Quick*-RNA Faecal/Soil Microbe Microprep kit(Zymogen Research)	Z	+	++	−	+
NucleoBond RNA Soil Mini kit for RNA from soil(Machery-Nagel)	Nm	+	+	+	+
NucleoBond RNA Soil Midi kit for RNA from soil(Machery-Nagel)	nd	++	−	+	--
RNeasy PowerSoil Total RNA kit(Qiagen)	Q	++	++	+	+

### Sequencing data

**Fig. 2.**
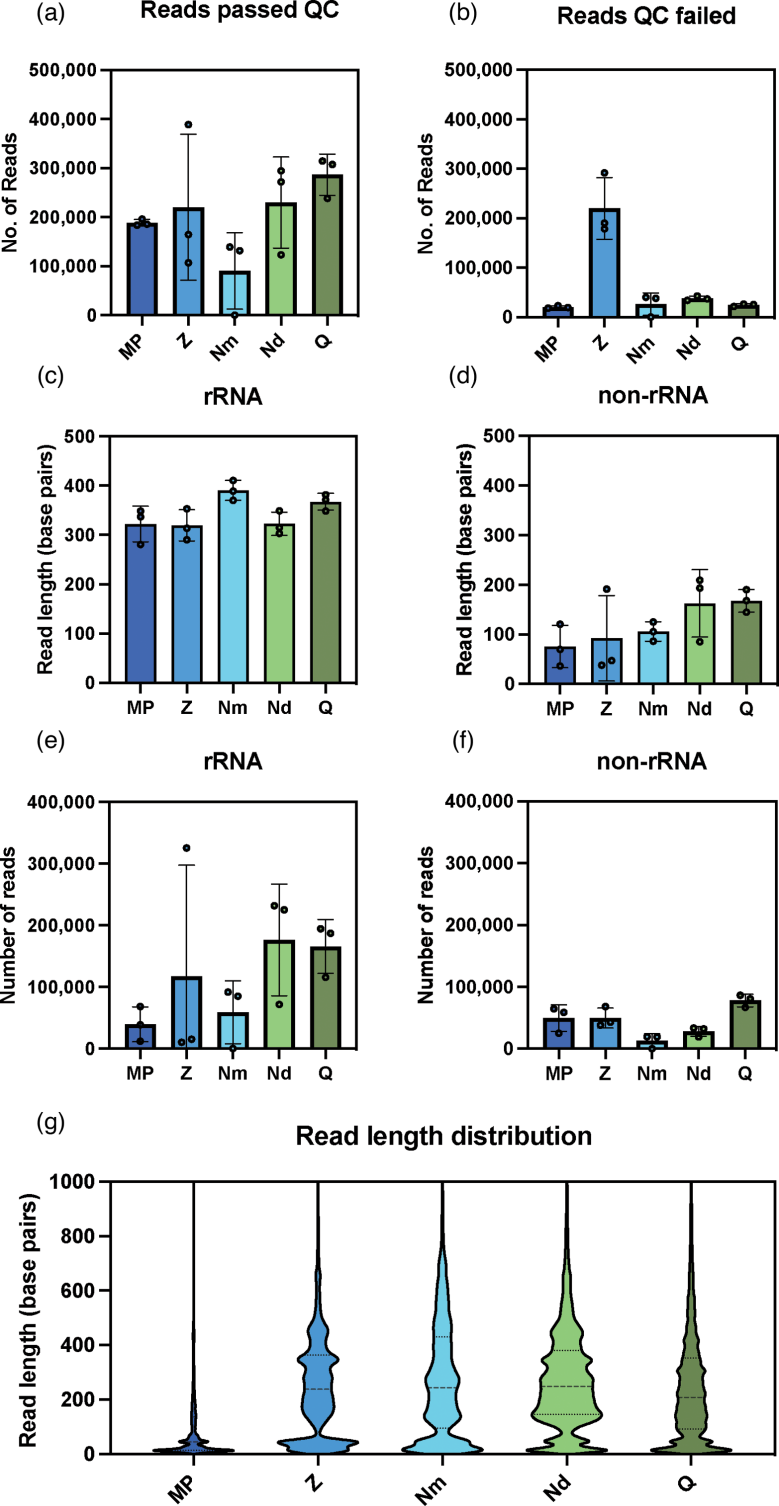
Analysis of RNA sequence data from commercial kits. The number of reads that passed quality control during sequencing on a MinION flow cell (**a**) and the number of reads which failed (**b**) was quantified for libraries prepared from each extraction kit, MPBio (MP), Zymo (Z), Nucleobond Mini (Nm), Nucleobond Midi (Nd), and Qiagen (Q). The average length (**c-d**) and number of the reads (**e-f**) that were sorted into rRNA and non-RNA transcripts was assessed. Distribution of read lengths from all sequence data produced from each extraction kit was also calculated (**g**). Error bars represent SD. *n*=3.

The number of reads that passed with a Phred quality score of greater than nine for ONT sequencing was quantified (Fig. 2a) with the Q kit displaying the fewest number of failed reads per read that passed (Fig. 2b). Notably the Z kit displayed an almost equal number of reads that both passed and failed to reach the quality threshold. All reads which passed the quality filtering were sorted using sortmerna [25] into successfully aligned reads and unaligned, denoted as ‘rRNA’ and ‘non-rRNA’ respectively and further characterised. In the RNA sorted fraction, the Nm kit gives the longest average read length (Fig. 2c), however the Nd kit yields the greatest average of total reads (Fig. 2e). In the non-rRNA proportion, the Q kit gives the longest average read length for that category (Fig. 2d) and the largest proportion of reads that fall into that category (Fig. 2f). Read length distributions are shown for each of the kits (Fig. 2g). The majority of reads from each kit are below 1000 base pairs with the Nm, Nd and Q kits having a greater number of reads between 500 and 800 base pairs than the MP and Z kits. The number of reads produced by each kit greater than 600 base pairs in length, the maximum achievable with paired end short-read Illumina sequencing, was also quantified (Table S1). The Q kit had the greatest number of reads over 600 base pairs. As a percentage of total reads the Nm, Nd and Q all had a significantly greater proportion of reads over 600 base pairs than the MP and Z kits (Table S1). An ANOVA and subsequent Tukey’s multiple comparisons test showed no significant difference was observed in the proportion of reads over 600 base pairs between the Nm, Nd and Q kits (*P*<0.05).

**Fig. 3.**
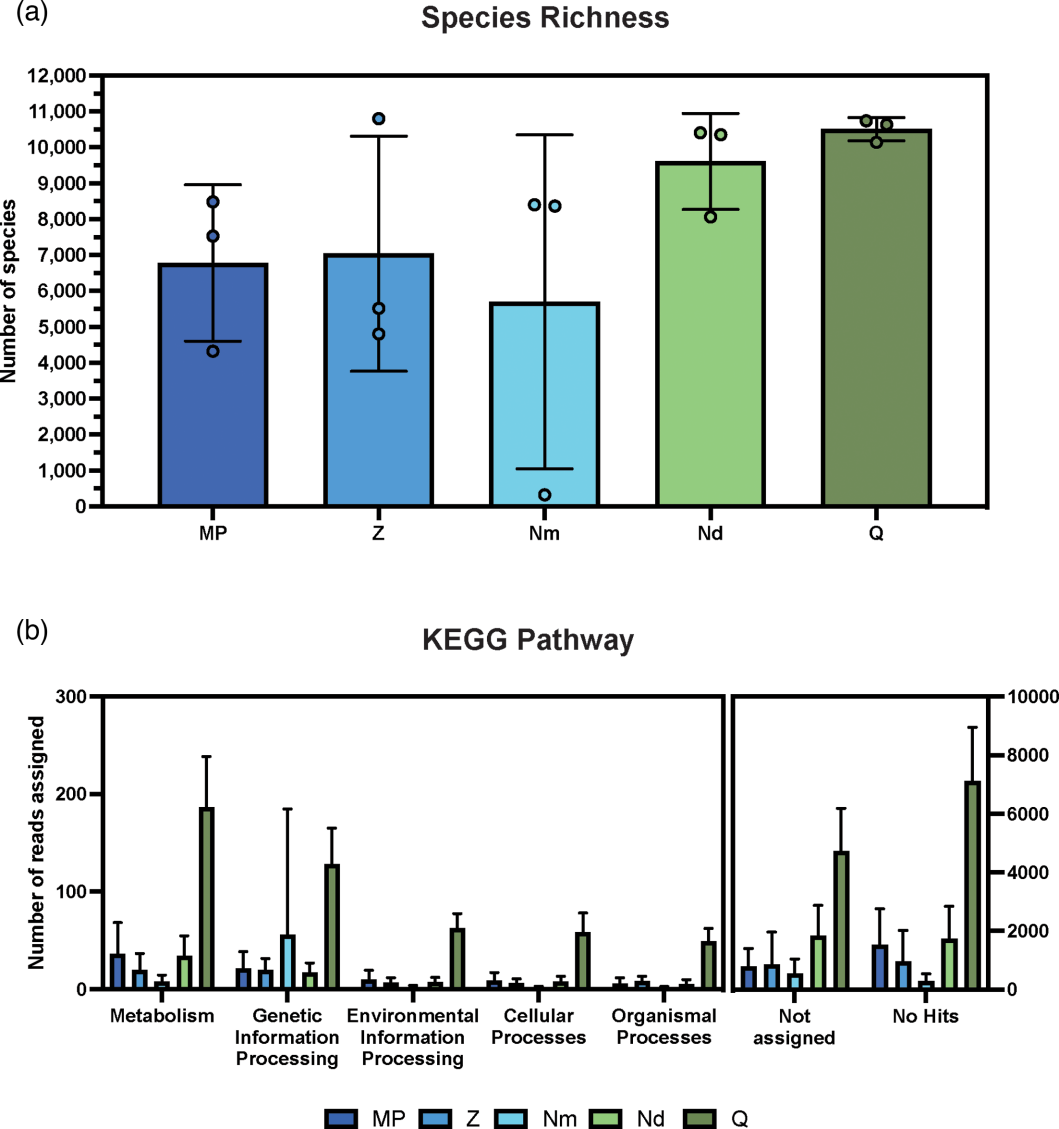
Species richness and functional gene assignments for each extraction kit. Species richness was calculated for each of the samples after classification of all reads from each sample sequenced on a MinION flow cell (**a**), MPBio (MP), Zymo (Z), Nucleobond Mini (Nm), Nucleobond Midi (Nd), and Qiagen (Q). After filtering rRNA from the data, non-rRNA reads were classified and the number of reads from each sample assigned to broad functional groups in the KEGG database were quantified (**b**). Error bars represent SD. *n*=3.

Depth of sequencing was assessed using rarefaction curves derived from the classification of all sequence reads at species level (Fig. S1, available in the online version of this article). Species richness was determined for each sample (Fig. 3a). An ANOVA with Tukey multiple comparisons showed that significant differences were only observed between the Q and Nm kits (*P*<0.05). The Q kit produced the highest average of 10 506 species in the samples and had the lowest standard deviation in species richness of all kits. Reads sorted as ‘non-rRNA’ were classified against the NCBI nr database using DIAMOND. The number of reads assigned to top order pathways in KEGG were quantified for each kit as were the number of reads reported as ‘Not assigned’ and ‘No Hits’ (Fig. 3b). Low read counts were observed for all the samples in each of the selected functional categories, with the Q kit providing the highest number of reads in the metabolism category at an average of 187. The number of reads reported as ‘Not assigned’ and ‘No hits’ was much greater than any of the functional categories for all kits.

### Consistency across soil types

**Fig. 4.**
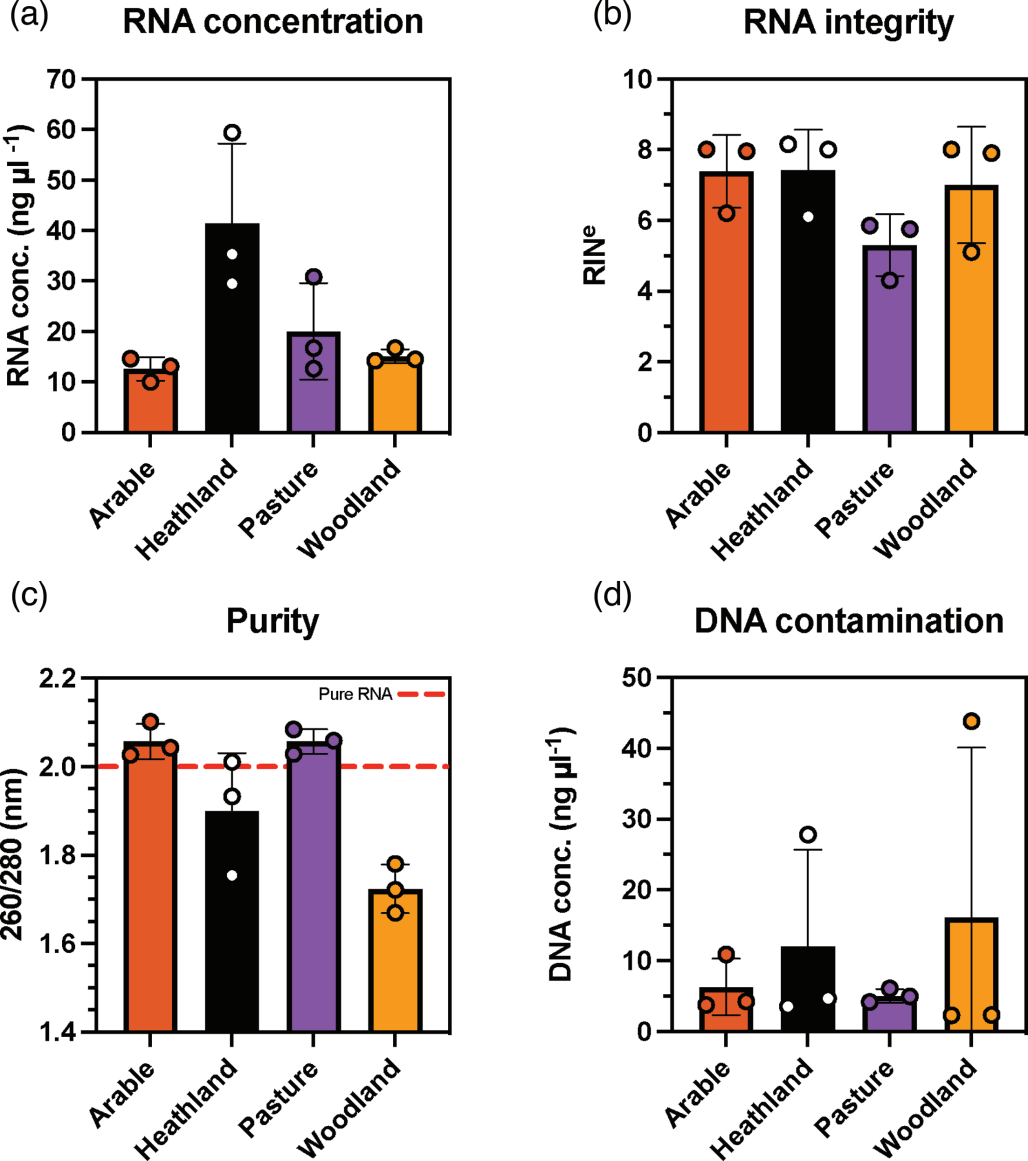
RNA extraction from different soil types RNA concentration (**a**), integrity (**b**), purity (**c**) and DNA contamination (**d**) were quantified for replicate extractions performed using the Q kit on arable (red), heathland (black), pasture (purple) and woodland (yellow) soil. Error bars represent SD. *n*=3.

To assess performance across different land uses, soil samples were collected for RNA extraction using the Q kit as this kit extracted the highest quality RNA samples overall. An ANOVA with subsequent Tukey multiple comparisons displayed significant differences (adjusted *P*<0.05) in RNA concentrations between the woodland and heathland soils as well as between the arable and heathland soils (Fig. 4a). RNA concentration was highest in the heathland soil with an average concentration of 41.4 ng µl^−1^, however each soil type had sufficient yield for multiple ONT cDNA library preparations. RIN^e^, displayed as the mean of scores generated in Eukaryote and Prokaryote mode, are consistent between the arable, heathland and woodland soil with average RIN^e^ scores of 7.4, 7.4 and 7 respectively. The pasture soil displayed lower RNA integrity with an average RIN^e^ of 5.3 (Fig. 4b). However no significant differences in RIN^e^ were found between any of the soil types. Purity was calculated for each sample (Fig. 4c) and showed the two agricultural soil types, arable and pasture, both had high average purity scores of 2.1. Of the two natural soils the heathland had the better average purity score of 1.9 with the most highly DNA contaminated sample for that soil type having the lowest purity score of all heathland replicates. The woodland soil was the least pure of all soil types with an average score of 1.7. DNA contamination was quantified for each soil type (Fig. 4d), the highly organic heathland and woodland samples had the highest levels of DNA contamination with average concentrations reaching 12 ng µl^−1^ in the heathland samples and 16.2 ng µl^−1^ for the woodland. The purity of all the samples was also quantified after DNase treatment, PolyA tailing and bead purification. These processes improved purity scores based on 260 : 280 ratios as well as 260 : 30 (Fig. S2).

**Fig. 5.**
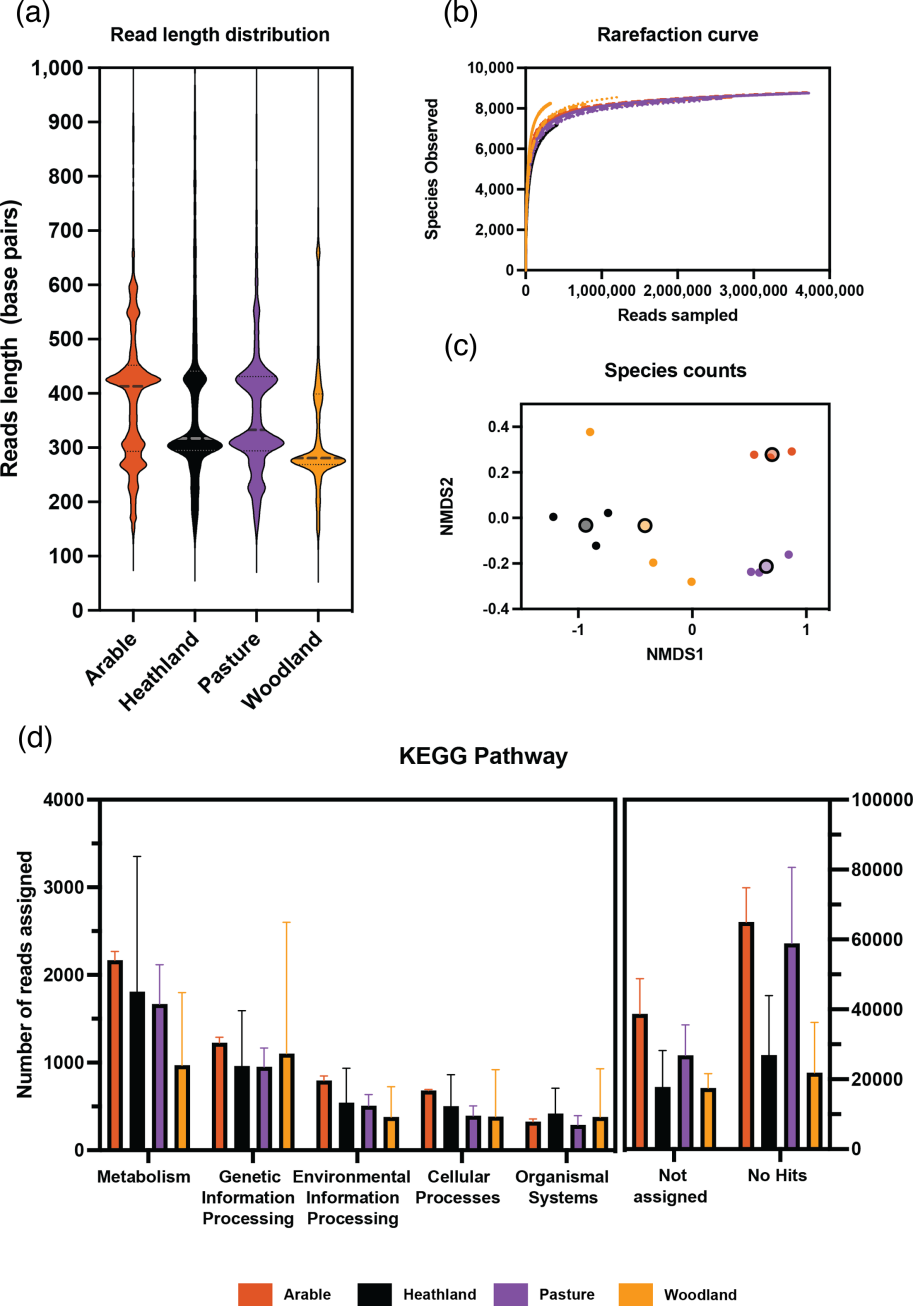
RNA sequence read analysis across different soil types. Read length distributions of all sequenced samples for each soil type, arable (red), heathland (black), pasture (purple) and woodland (yellow) soil, was calculated after sequencing on a PromethION flow cell (**a**) and saturation curves based on species counts extracted from the classification of all reads were produced (**b**). NMDS based on Bray-Curtis distances calculated from species counts (**c**) show clear separation of samples by soil type (ADONIS2, *P*<0.05). All reads were sorted using sortmerna and the number of non-rRNA reads classified into broad KEGG categories for each soil type was quantified (**d**). Error bars represent SD. *n*=3.

Distribution of read lengths that passed quality control after sequencing using a PromethION flow cell differed between soil type (Fig. 5a). The arable soil had the largest proportion of reads between 400 and 600 base pairs. Rarefaction curves (Fig. 5b) show that some samples were sequenced to saturation based on species classification of all sequence reads. NMDS of the ecological distances between each of the samples derived from the species counts was calculated (Fig. 5c). PERMANOVA analysis using ADONIS2 displayed significant separation (*P*<0.05) of soil type centroids, with the greatest dispersion of replicates found in the woodland and heathland soils. Sequence data was processed as before by sorting transcripts into ‘rRNA’ and ‘non-rRNA’. The ‘non-rRNA’ reads were classified against the NCBI nr protein database using DIAMOND. The number of reads that were classified into top order pathways in KEGG (Fig. 5d). The arable soil had the largest number of reads that classified to each of the functional groups with 5190 in total. Across all the soil types, there were 17 217 reads mapping to metabolism genes.

## Discussion

### Comparison of RNA yield and quality from five extraction kits

Our data shows the Qiagen RNeasy PowerSoil Total RNA kit (Q) is the most effective at extracting high quality RNA molecules from soil at sufficient yield for total RNA sequencing. The diverse and complex matrix exhibited by the soil environment typically yields low concentration, low quality and impure RNA extractions [13] which can present a barrier to effective metatranscriptomic analysis [11]. We demonstrate that the Q kit extracts high concentrations of more intact RNA transcripts that are free from sample matrix impurities (Fig. 1). While the Q kit provided the overall highest quality RNA extractions, extraction time is one of the longest at approximately 5–7 h depending on the length of optional incubation steps and number of samples processed. The Z kit has a fast extraction time of approximately 2 h with a higher throughput than the Q kit facilitated by the use of mini-spin columns, it also had the highest average RIN^e^ of all the raw extractions. The poor sequencing results displayed by the Z kit are likely a result of the low purity of these samples, as humic acids interfere with PCR and exonuclease steps during sequencing library preparation [30]. Soil samples could undergo a pre-treatment step to reduce humic acid content in the soil [31] and therefore the Z kit may become a more appropriate option for RNA extraction from soil. However, the Z kit has a low elution volume and RNA concentrations could be affected by any additional processing to increase purity. These factors make the benefits of the Z kit ‘out of the box’ insufficient for it to be recommended over the Q kit.

### RNA yield and quality across different sample types

Commercial RNA extraction kits enable standardisation between laboratories and increase sample throughput due to process optimisation by the manufacturer. Furthermore, the number of individual samples that need to be processed to achieve sufficient replication to perform robust differential expression analysis make consistency key to metatranscriptomic analysis in soil [10]. Commercially available RNA extraction kits enable these required consistencies and efficiencies, but are considered limited in their application due to proprietary buffer recipes [32]. Custom RNA extraction protocols enable refinement and are suggested to be more appropriate across different soil types [14]. However, we found that the Q kit was consistent between several different soil types with identical extraction procedures. Samples with high organic contents, such as, the heathland and woodland soils (Table S2), did require careful attention. They had a more poorly defined biphasic layers after lysis in phenol-chloroform and both also required increased time to clarify through gravity purification columns, with some samples clogging the membranes. A small adaptation of reducing soil input weight could improve extraction efficiency for highly organic samples without the need for other alterations to the extraction procedure.

### Challenges of long-read metatranscriptomic analysis of soil samples

Between each of the kits tested, the Q kit provided total RNA samples that gave the greatest number of transcripts putatively identified as mRNA when filtered using sortmerna. Further analysis showed that the Q kit had the longest average read length of putative mRNA sequences (Fig. 2), demonstrating a greater ability to maintain the originating transcript length compared with the other kits trialled in this study. The number of reads that are successfully assigned to functional genes however, was still relatively low even for the Q kit. The depth of coverage achieved from a single sequencing run using ONT is clearly a factor in the presence of low read counts for transcripts classified at gene level. ONT offer a range of sequencing flow cells, with MinION flow cells outputting up to 50 Gb of data and PromethION flow cells capable of producing 290 Gb of data. This difference in potential data output contributed to the increase in the number of transcripts assigned to KEGG functional groups between samples sequenced on a MinION flow cell and those sequenced using a PromethION flow cell (Figs3b  5d). Multiplexing 15 prepared RNA sequencing libraries from soil samples on MinION flow cells did not reach sufficient sequencing depth for robust metatranscriptiomic analysis (Fig. 3). However, multiplexing 12 samples on a PromethION flow cell increased sequencing depth (Fig. 5) but coverage of mRNA transcripts was still insufficient for differential analysis. Ribodepletion can be used as an approach to enrich the mRNA fraction of soil samples [14], this study however used total RNA samples for sequencing library preparation. Total RNA analysis requires sufficient sequencing depth to achieve robust differential expression analysis of the mRNA fraction, as greater than 80% of total RNA in a cell may be rRNA [33]. The high proportion of rRNA in total RNA samples is considered a barrier to differential expression analysis as it is uninformative in elucidating important molecular pathways in response to an experimental condition [34]. As RNA samples from soil already suffer from low yield this makes ribodepletion an attractive approach to enrich mRNA sequences but could also incur additional loss of the already low concentration of informative sequences [14]. In addition, due to the heterogeneity of the soil community, ribodepletion techniques that can effectively cover all taxa in the sample is challenging and expensive [35]. Moreover, rRNA sequences from soil samples can be informative in identifying the active microbiome of soil environments [36]. This data may be complementary to soil studies that employ bulk measurements of soil microbial activity [37] by identifying the abundance of taxa contributing to respiration and microbial biomass. The development of more cost effective and targeted approaches to ribodepletion for soil metatranscriptomic studies, such as those applied in laboratory culture [38], could help to make rRNA depletion of total RNA samples from soil more viable for improving the coverage of mRNA transcripts in the sequencing data.

Another potential solution to the low sequencing coverage of mRNA transcripts would be to multiplex fewer samples per flow cell or perform more sequencing runs for each sample. This approach may be cost prohibitive due to the consumables associated with RNA library preparation and sequencing, so maximising pore occupancy on the flow cell by optimising loading concentrations for soil samples could improve the yield of mRNA reads determined after filtering with sortmerna. However, increases in the number of reads reported as ‘Not assigned’ and ‘No Hits’ also increased with higher read counts generally in this study (Fig. 3b). This indicates that a large proportion of sequences are not represented in the KEGG database, a problem not solvable through increasing sequence depth with additional runs or higher throughput flow cells [39].

Another challenge with environmental studies are that reference databases typically have a clinical focus, and therefore underrepresent taxa and pathways of diverse taxa [39]. Increasing the characterisation of novel species and genes from soil microbes in available databases would reduce the number of reads with ‘No Hits’ and requires a concerted effort from the field of soil microbiology, an endeavour that may be improved by the adoption of ONT in soil science as an accessible sequencing technology. Species richness of the active microbiome was not greatly affected by sequencing depth as both MinION flow cell and PromethION flow cells produced similarly saturated rarefaction curves (Figs 5b and S1). Extraction kit selection similarly did not have a significant impact on observed species richness for the same soil sample (Fig. 3a). In general, a greater number of species was observed from kits which extracted a higher quantity and quality of RNA from soils. This similarity between kits and flow cell choice in characterising the transcriptionally active microbiome implies that several of the extraction kits may be viable for gathering data of this kind. Considerations such as cost and throughput associated with the protocol of each extraction kit may be more important in these circumstances.

Finally, this study found that while the Q kit provided an average of 23 325 reads over 600 base pairs, and 10 800 reads longer than 800 base pairs, most sequence reads were of a length appropriate for short-read sequencing. With the average length of a bacterial gene around 1000 base pairs [40] and paired end Illumina reads reaching 600 base pairs it could be argued that long-read sequencing for the samples in this study did not provide a significant benefit. However, while obtaining full length transcripts for a large proportion of some soil samples may be unrealistic, the inclusion of long reads in the assembly process may improve the quality and length of the resulting contigs [41]. Optimising PCR conditions and number of cycles as well as adapting the size selection step post-PCR barcoding may help to increase the number of long reads during ONT sequence library preparation. However, there is also a danger that aggressive size selecting in the pursuit of long reads may exclude short RNAs of interest.

## Conclusion

The extraction of good quality RNA at high yields is replicable across soil types using the Qiagen RNeasy PowerSoil Total RNA kit. Oxford Nanopore Technology sequencing has the potential to offer a robust sequencing platform for soil metatranscriptomics. However, specific adaptation of library preparation methods is required to maximise the benefits of long-read sequencing for total RNA extracted from soil. Clear improvements in read length and sequencing depth are achievable through adaptation of the current library preparation methods, making the pursuit of long-read metatranscriptomics in soil a worthwhile endeavour. The potential to capture full length transcripts from the complex soil environment is very attractive for the wide range of soil processes that would greatly benefit from the insight this data could yield.

## supplementary material

10.1099/mgen.0.001298 Uncited Supplementary Material 1.

## Abbreviations

BR: Broad Range
HS: High Sensitivity
KEGG: Kyoto Encyclopedia of Genes and Genomes
mRNA: Messenger RNA
NCBI: National Centre for Biotechnology Information
NGS: Next Generation Sequencing
NMDS: Non-metric MultiDimensional Scaling
ONT: Oxford Nanopore Technologies
PCR: Polymerase Chain Reaction
RIN^e^: RNA Integrity Number
rRNA: Ribosomal RNA
