# Supplementary material for: Evaluation of commercial RNA extraction kits for long-read metatranscriptomics in soil
Source: Microb Genom. 2024 Sep 19;10(9):001298. doi: 10.1099/mgen.0.001298 (PMC11412367; doi:10.1099/mgen.0.001298)
Supplement: Uncited Supplementary Material 1. [file mgen-10-01298-s001.pdf]

Evaluation of Commercial RNA Extraction Kits for Long-Read Metatranscriptomics in Soil

Supplementary information

Daniel G. Barber [a], Christian A. Davies [b], Iain P. Hartley [a] and Richard K. Tennant [a].

Corresponding Author:
R.K.Tennant@exeter.ac.uk

Kit	Total Reads					
MP	189958	197598	212329	176852	173534	179614
Z	106771	164507	388526			
Nm	323	145526	150261	344	117049	127568
Nd	134230	308595	285703	112108	280130	258183
Q	242878	310144	315732	233264	304095	313144
	Reads over 600 basepairs					
MP	11057	5529	1237	11479	5557	1272
Z	1485	1234	31461			
Nm	32	17149	14103	33	12811	12245
Nd	13488	17115	18347	13488	17115	18347
Q	16105	22045	28447	18264	23716	31375
	Reads over 800 basepairs					
MP	6972	3307	728	7382	3278	734
Z	769	664	14297			
Nm	12	5838	4429	17	4406	4004
Nd	5041	6878	8198	4250	6605	7826
Q	7382	10208	13077	8464	11010	14663

Supplementary Table 1. Quantification of long reads

Soil type	Dominant plant species	Sampling depth (cm)	Soil pH	Organic matter content (%)	Carbon content (%)	Field capacity (%)
Arable	Spring Triticale	0-10	5.91	3.66	2.12	26.51
Heathland	Common heather (Calluna vulgaris)	0-10	3.87	52.57	30.49	48.34
Pasture	Mixed grass (dominant Ryegrass Lolium perenne)	0-10	6.17	5.27	3.04	35.41
Woodland	Oak (Quercus robur) and Beech (Fagus sylvatica)	0-10	3.5	12.95	7.51	40.88

Supplementary Table 2. Soil characteristics for Clinton Estate samples

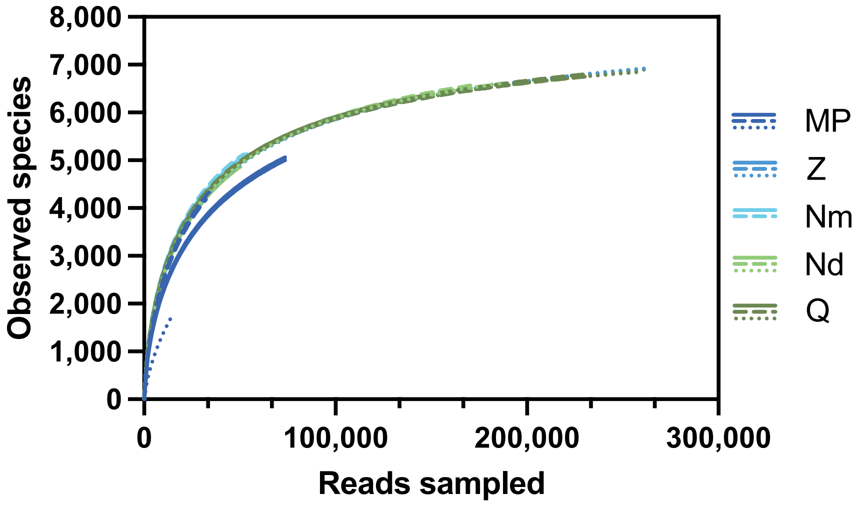

Supplementary Figure 1: Rarefaction curves

Saturation curves based on species counts generated on a MinION flow cell between each RNA extraction kit tested.

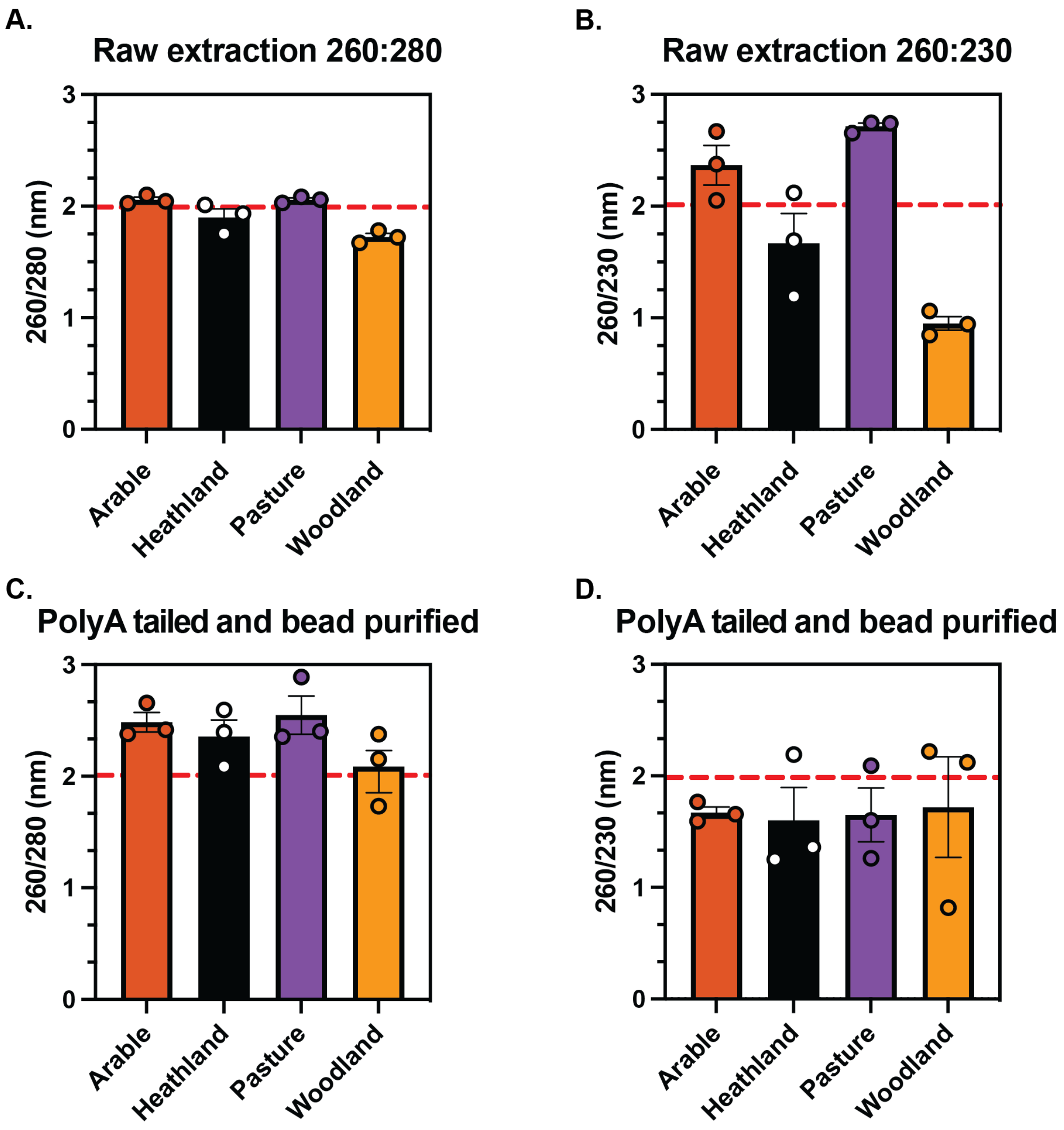

Supplementary Figure 2: Effect of bead purification on RNA purity.

Quantification of purity metrics for RNA extracted from different soil types using the Q kit before and after PolyA tailing and bead purification.
